# Multi-Year Impacts of Ecotourism on Whale Shark (*Rhincodon typus*) Visitation at Ningaloo Reef, Western Australia

**DOI:** 10.1371/journal.pone.0127345

**Published:** 2015-09-23

**Authors:** R. L. Sanzogni, M. G. Meekan, J. J. Meeuwig

**Affiliations:** 1 School of Animal Biology (Oceans Institute), the University of Western Australia, 35 Stirling Hwy, Crawley, Western Australia, 6009, Australia; 2 Australian Institute of Marine Science c/o UWA Oceans Institute (MO96), 35 Stirling Highway, Crawley, Western Australia, 6009, Australia; 3 Centre for Marine Futures (Oceans Institute), the University of Western Australia, 35 Stirling Hwy, Crawley, Western Australia, 6009, Australia; University of California Davis, UNITED STATES

## Abstract

In-water viewing of sharks by tourists has become a popular and lucrative industry. There is some concern that interactions with tourists with ecotourism operations might harm sharks through disruption of behaviours. Here, we analysed five years of whale shark (*Rhincodon typus*) encounter data by an ecotourism industry at Ningaloo Reef, Western Australia, to assess the impact of ecotourism interactions on shark visitation, within the context of the biological and physical oceanography of the region. Our data base consisted of 2823 encounter records for 951 individual whale sharks collected by ecotourism operators between 2007 and 2011. We found that total encounters per whale shark and encounters per boat trip increased through time. On average, whale sharks re-encountered in subsequent years were encountered earlier, stayed longer and tended to be encountered more often within a season than sharks that were only encountered in a single year. Sequential comparisons between years did not show any patterns consistent with disturbance and the rate of departure of whale sharks from the aggregation was negatively correlated to the number of operator trips. Overall, our analysis of this multi-year data base found no evidence that interactions with tourists affected the likelihood of whale shark re-encounters and that instead, physical and biological environmental factors had a far greater influence on whale shark visitation rates. Our approach provides a template for assessing the effects of ecotourism interactions and environmental factors on the visitation patterns of marine megafauna over multiple years.

## Introduction

Ecotourism has become increasingly popular over the past 20 years and is a rapidly growing sector within the tourism industry [1]. Marine environments support a range of ecotourism attractions, often providing opportunities to view iconic megafauna at close range [2–4]^.^. Shark diving, defined here as the in–water viewing of sharks in their natural habitat, is one of these activities[5]. The economic benefits of shark diving can be considerable [3,6] and provide a compelling argument for conservation of shark species, as well as an alternative to extractive practices like fishing [6–9]. For this reason, shark diving is considered an integral part of conservation strategies for these animals [5].

Whale sharks (*Rhincodon typus*) are a major target of the ecotourism industry [3,10]. These sharks are valued highly by the industry because of their size (the world’s largest fish), body colouration, relatively harmless nature (they are plankton-feeders) and importantly, because they form predictable seasonal aggregations in coastal waters throughout the tropics [11–12]. These traits make them a popular, accessible and reliable target for ecotourism. Interactions with whale sharks generally take the form of “swim-with” encounters where tourists are permitted to snorkel near the animals [13]. This form of ecotourism provides a strong economic incentive for protecting a species that is rare and vulnerable [14–16].

Whale sharks exhibit slow growth and reach maturity at a late age [14,16–18], traits that render them vulnerable to human impacts. Indeed, until recently, whale sharks were hunted in various parts of their range including India [19], the Maldives [7], and Taiwan [20]. Additionally, whale sharks have been captured intentionally by purse seine fishing vessels because fish that are target species, such as tunas, tend to aggregate around the sharks [21]. These impacts combined with their relative scarcity, have led to the classification of whale sharks as “Vulnerable” on the International Union for the Conservation of Nature (IUCN) Red List of Threatened Species [22]. While whale shark ecotourism seems like a sustainable alternative to extractive practices, there is some evidence that interactions with tourists may negatively impact animals. At Gladden Spit in Belize, whale shark encounters declined between 1998 and 2003, a pattern suggested to be linked to an increase in the number of divers across the same period [23]. Furthermore, responses such as rolling, banking, and diving in response to approaches by divers and boats have been recorded at a number of different localities and are argued to be negative reactions to the presence of humans [15,23–25].

Whale sharks, the majority of which are immature males, aggregate at Ningaloo reef in Western Australia, from March to July with approximately 300 to 500 sharks visiting Ningaloo in a given year to feed on zooplankton [26]. The Ningaloo aggregation was one of the first to be a focus of ecotourism. After its inception in 1989, the industry grew swiftly from 1993 and was estimated to be worth approximately AU$6 million in 2006 [13,27–29]. At Ningaloo, there are some suggestions that whale sharks have become wary of ecotourism vessels and that the mean swimming distance between sharks and vessels increases over the course of a season [24]. Additionally, concerns have been raised about the potential for injury to the sharks from boat strikes [30]. However, long-term visitation patterns have not yet been linked to tourism encounters and studies of encounters of sharks with tourists have only assessed short-term (minutes to hours) behavioural responses. At present there is no empirical evidence that these interactions result in cumulative impacts on sharks over seasons or between years that could lead to them avoiding aggregation sites.

The Western Australia Department of Parks and Wildlife (DPaW) has implemented a number of measures to manage encounters between operators, tourists and whale sharks, thereby mitigating potential impacts on the animals. Restrictions are in place that limit the number of operator licences and a code of conduct for ecotourism was developed in 1995 [13,27]. The code stipulates that swimmers may not touch or ride sharks, approach closer than 3 m from the head or body, use underwater flash photography during encounters, or utilise SCUBA equipment or “motorised propulsion aids” [27]. Additionally, DPaW carry out regular flights over the aggregation area during the season to monitor compliance to the code (Emily Wilson, DPaW pers. comm). This code has been adopted by management agencies and operators in a number of other locations world-wide [15,25,31].

The aggregation of whale sharks at Ningaloo Reef offers a unique opportunity to assess potential long-term (multi-year) impacts of ecotourism as animals here are exposed to interactions that occur in a standardised manner governed by the code of conduct. Furthermore, a long term photographic data base of encounters has been collected by DPaW. Because whale sharks can be individually identified from these photographs [32], encounter records for animals can be constructed from the data base over multiple years and can then be used to quantify the impacts of ecotourism on whale shark visitation rates.

Here, we examine the impact of ecotourism encounters on whale shark visitation using a five year encounter data set focussing on the years 2007 to 2011. We utilised a set of metrics that are applicable to any species where animals can be identified individually and multi-year encounter records exist.

## Methodology

### Study site and aggregation

Photographic images of whale sharks were collected from 2007 to 2011 at Ningaloo Reef, a fringing coral reef situated along the northwest coastline of Western Australia (for location map see Fig 1 in Meekan et al. 2006). Whale sharks aggregate at Ningaloo Reef annually from March to July [33]. Around 80% of these sharks are juvenile males that average between four and five metres in total length [26]. Maturity in male sharks is determined by the size of claspers and for this population maturity occurs at around 8 metres total body length [34].

**Fig 1:**
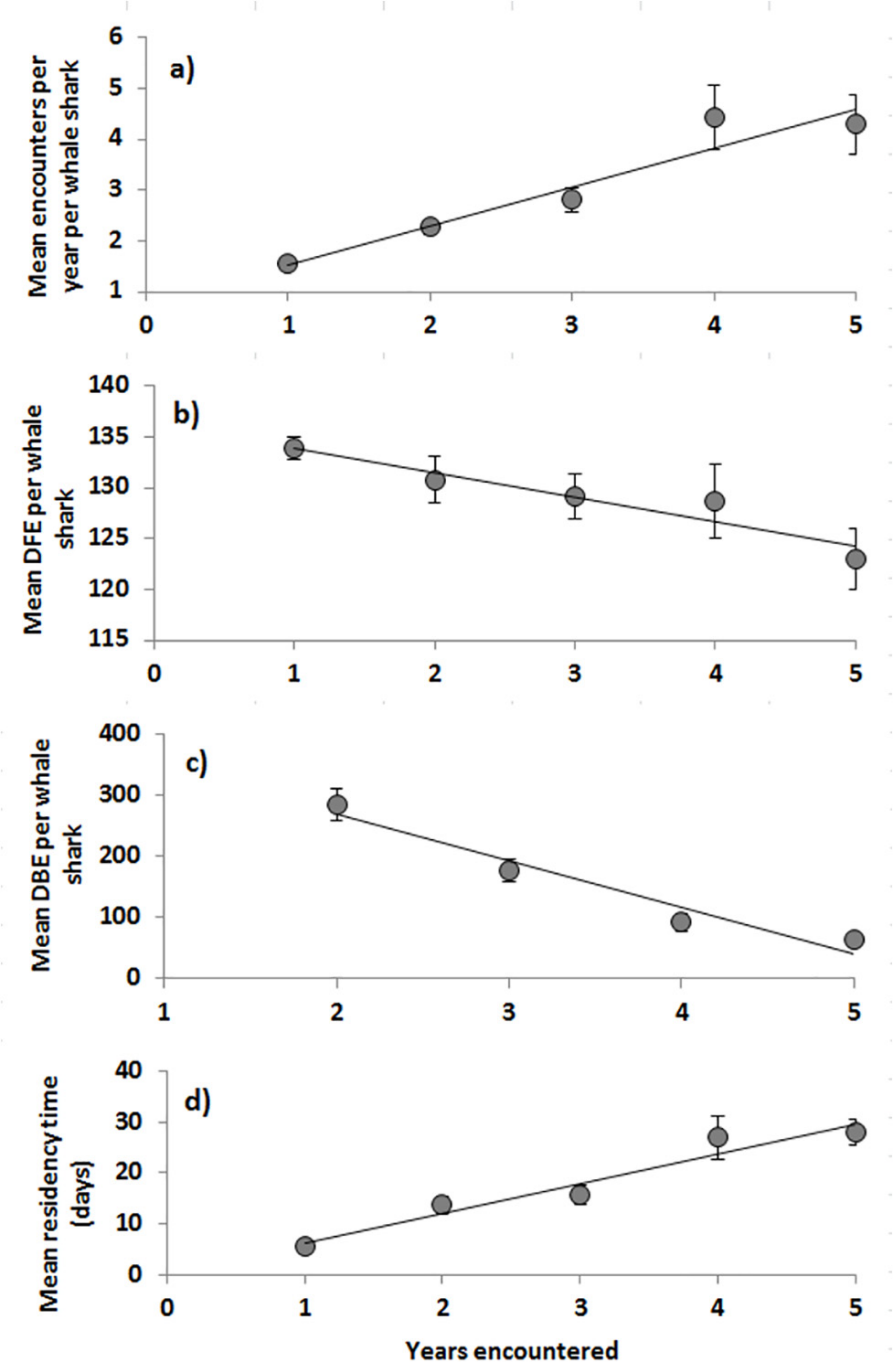
Relationships between encounters and the number of years an individual whale shark was observed for a) mean encounters per year per whale shark, b) mean day of first encounter per whale shark and c) mean days between encounters (DBE) per whale shark and d) mean residency time per whale shark. Standard errors (SE) calculated from individual sharks. 1a) Mean encounters = 0.77years + 0.77, R^2^ = 0.92, p = 0.009; 1b) Mean day of first encounter = -2.4years + 136.3, R^2^ = 0.91, p = 0.012; 1c) Mean days between encounters = 75.38years + 418.24, R^2^ = 0.95, p = 0.012; 1d) Mean residency time = 5.82years + 0.56, R^2^ = 0.94, p = 0.006.

### Whale shark ecotourism and interactions

The whale shark ecotourism industry currently involves a total of 14 licences to operators from DPaW to conduct whale shark tours. Spotter planes are used to locate sharks and communicate their position to the tour vessels. According to the DPaW code of conduct, when vessels encounter a whale shark a maximum of 10 snorkelers can enter the water to swim with the animal. Encounters can occur for as little as five minutes, but cannot continue for more than 90 minutes. Snorkelers must not approach closer than three metres and vessels must remain 30 m away from sharks. Only one operator may interact with a shark at a time [35] and each operator makes one boat trip per day (Emily Wilson, DPaW pers. comm). Due to multiple operators using the same spotter planes, sequential encounters by operators on the same shark are commonplace. Operators record every encounter in a data base that is maintained by DPaW.

A trained videographer is present to record encounter data on every trip during the entire whale shark season. During encounters, the videographer takes images of sharks with an underwater digital video or still camera [26]. Whenever possible, videographers take images of the right and left sides of each shark and record the sex and an estimate of size. These data are provided by all operators to DPaW on a monthly basis. Males are identified by the presence of claspers [14] and where sex could not be determined because a shark swam away or dived, individuals are recorded as indeterminate [26].

### Identification and matching of whale shark images

Sharks were identified individually based on the unique patterns of white dots and stripes present on their dorsal surface [26,36]. There is good evidence from re-encounters of sharks at more than decadal intervals that these markings do not change over time [26,32,37]. Identification images consisted of photographs of spot and stripe patterns in a standard area between the fifth gill slit and the edge of the pectoral fin [32]. These were matched in the photograph database using the Interactive Individual Identification System program (I^3^S) [38], which highlighted conspicuous spots on an image and then used an algorithm to compare dot patterns (“fingerprints”) among different images [38]. Final matches were confirmed by eye (for details see: http://www.reijns.com/i3s/). The dataset produced from the matching process contained a list of every individual shark seen between 2007 and 2011, the day of the first encounter and subsequent re-encounters for each shark, the flank the image was recorded from, gender and size of the shark and the ecotourism operator that recorded the image. Although large, our database did not contain records from all encounters by the tourism industry that occurred in a year. Typically, we received data from seven to 10 operators per year with some only reporting encounters from a majority of their total boat trips. Additionally, some images (approximately 20%) were excluded from the analysis as they were of poor quality (incorrect angle, poor visibility, not centred on reference areas etc.) and could not be used for matching. Our database also included photographs provided by the Australian Institute of Marine Science (AIMS) that were collected during annual research trips to Ningaloo Reef for two weeks at the height of the season every year of the study period.

### Indicators of whale shark response to interactions

Because a whale shark cannot be photographed and identified without the presence of a snorkeler in the water, we could not compare reactions of the sharks with and without the presence of an observer. However, we did have a gradient of single to multiple tourist interactions with sharks. If tourism encounters were having a negative impact on sharks we anticipated that the total encounters per shark would decline, the day of first encounter would occur later each season, the days between encounters within a season would lengthen, residency time would decrease and the rate of departure should increase. Given these hypotheses, we used several metrics to determine if encounters with tourism vessels affected the future visitation of whale sharks. For each identified shark, these were: (1) total encounters per shark per year (TE); (2) the day of first encounter per year (DFE); (3) the mean number of days between within-year encounters per year (DBE); (4) mean residency time per year; and (5) the annual rate of departure from the aggregation for all sharks. Residency time was defined as the length of time between the first encounter with a whale shark and the last in any given year. To calculate the rate of departure we fitted a linear regression between the peak of monthly total encounters during a season (May) and the commencement of the end of season, when encounters plateaued at low values (July). The slope of this relationship was used as an indicator of the rate at which whale sharks left the aggregation. We compared total encounters, day of first encounter and days between encounters, between consecutive years for the entire study period in relation to earlier exposure to interactions (total encounters per year).

We also examined the relationship between whale shark encounters per boat trip and the total number of trips in order to determine any effect of variation in sampling effort. These data were collected from seven to 10 operators per year and included data collected on AIMS research trips to Ningaloo Reef. All operators provided data each year however, the quantity of data varied due to some operators not using their licences in particular years, and the addition of an operator in both 2009 and 2011. Encounters per trip were calculated as the total number of encounters per month divided by the number of boat trips reporting encounters (BT_R_). This effort estimate (BT_R_) is not equivalent to the number of encounters, because vessels would only make one trip per boating day, but could encounter between one and 10 individual sharks per trip. The total number of boat trips per month (BT_TOT_) was also calculated based on records for all operators provided by DPaW, with BT_R_ thus a subset of BT_TOT_. Any difference between BT_R_ and BT_TOT_ occurred as a result of removal of poor quality images from the dataset and opportunistic nature of videography.

We considered it possible that technological creep may have occurred in the industry during the period of our study, where better techniques could lead to an improvement in locating sharks [39–40]. Therefore, it was possible that our calculated encounters per trip masked underlying declines in whale shark numbers. To this end, we calculated the number of whale sharks per spotter plane hour per year and compared this to encounters per trip to account for any potential technological creep. Spotter plane hours per year were calculated from annual records kept by DPaW.

There is evidence to suggest that the abundance and arrival times of whale sharks at Ningaloo are influenced by the environmental parameters of wind shear (WS), the Southern Oscillation Index (SOI), sea level (SL), sea surface temperature (SST) and chlorophyll *a* [32,41]. Consequently, we analysed our metrics in relation to these variables on a monthly and seasonal basis. Monthly Exmouth SL and SOI values were extracted from the Australian Bureau of Meteorology website (www.bom.gov.au). We calculated mean monthly SST and chlorophyll *a* values for an area bounded by the coordinates 22° to 23°S and 113° to 114° E directly off Ningaloo Reef using 4 and 9 k m^2^ blocks respectively. These data were provided by the SeaWiFS and Modis A satellites and extracted from the National Aeronautics and Space Administration Ocean Data website (http://www.oceandata.sci.gsfc.nasa.gov/). Monthly mean SST was calculated with MATLAB software using the approach of Cummins [42]. We calculated monthly wind shear values from 0.25° blocks within the area bounded by the same coordinates. These data were extracted from the National Climatic Data Center webpage for blended sea winds located on the National Oceanic and Atmospheric Administration website (http://www.ncdc.noaa.gov/oa/rsad/air-sea/seawinds.html). We applied a 60° clockwise transformation to V vector data using MATLAB software following the approach of Sleeman et al [41].

### Analysis of response to interactions

Total encounters, days between encounters, and day of first encounter per whale shark were averaged for each year (2007–2011). Analysis of variance (ANOVA) was used to test for differences in the key metrics among years; when ANOVA indicated there were significant differences among years, Tukey’s honestly significant difference (HSD) tests were used in order to identify the source of differences among years. Linear regression was used to analyse relationships between our metrics and independent variables including years of encounters, number of boat trips, SST, SOI, and wind shear (monthly and seasonal averages). All statistical analyses used the R software package (version 2.13.2) and Excel.

We tested if total encounters per whale shark differed between consecutive year pairs; with the prediction that sharks only encountered in year one would have higher encounters in year one than sharks that were re-encountered in year two if interactions disturbed the sharks at Ningaloo. We compared the number of sharks re-encountered between pairs of consecutive years using Welch’s t-tests with the expectation that any negative effect associated with interactions would lead to a decline in the number of individuals re-encountered the following year. Furthermore, we compared the residency time of re-encountered sharks of in year two with sharks only encountered in year two using Welch’s t-tests to account for possible inter-annual variability in shark behaviour.

To investigate the rate of departure from the aggregation, linear regression was used to analyse relationships between the departure rate, and total effort (boat trips) per year. We also used linear regression to investigate the relationship between inter-annual variability in boat trips (effort) and encounters per trip. We regressed trips per year against encounters per trip per year, and encounters per trip against the metrics of total encounters, days between encounters, day of first encounter, and whale sharks per spotter plane hour per year against encounters per trip per year. ANOVA was used to test for differences in encounters per trip among years. When ANOVA indicated there were differences between years, Tukey’s HSD tests were used to determine how years differed.

For the environmental analysis, we assessed the monthly variables of total encounters per whale shark, number of whale sharks, and whale sharks per trip using multiple linear regression with the environmental variables of wind shear (WS), the Southern Oscillation Index (SOI), sea level (SL), sea surface temperature (SST), and chlorophyll *a* concentration to determine whether environmental factors influenced visitation rates at Ningaloo. We also used linear regression to investigate annual trends in mean day of first encounter, mean days between encounters, total encounters per shark, encounters per trip, the slope of the rate of departure and total number of whale sharks against the environmental variables.

Our linear regression of mean days between encounters and the number of years a shark was encountered was confounded by the variable SST. Our environmental analysis revealed that SST was negatively correlated to days between encounters. In 2009 and 2010, SST was lower and days between encounters were higher than the other years. This artificially increased the mean DBE for sharks seen in four or five years as it was more probable they visited in 2009 and 2010. For this reason, we excluded data from 2009 and 2010 from this analysis and one year sharks as they were an outlier group.

## Results

### The Ningaloo whale shark aggregation

A total of 13 ecotourism operators and an AIMS research vessel made trips to encounter sharks over the five years of the study and whale shark imagery was gathered from seven to 10 operators per season. There were a total of 1608 boat trips across the five year study period where operators provided encounter data. The mean number of boat trips per year was 322 (± 37 SE, range 193–415). There were no significant differences in mean numbers of boat trips per operator per year (ANOVA p = 0.24, df = 4) with encounters per trip per year averaging 1.7 sharks (± 0.1 SE; range = 1.6–1.9) (Table 1).

**Table 1:** Descriptive statistics for the Ningaloo whale shark aggregation where DFE = the calendar day of first encounter per whale shark, DBE = days between encounters, EPT = encounters per boat trip and SST = sea surface temperature. All mean values are reported ± standard error (SE).

Year	Totalencounters	Totalsharks	MeanDFE (SE)	Mean DBE(SE)	EPT(SE)	Total boattrips	MeanSST (°C) (SE)
**2007**	309	134	141 (2.7)	2.5 (0.5)	1.6 (0.2)	671	26.2 (0.9)
**2008**	593	238	135 (1.7)	2.1 (0.3)	1.7 (0.1)	813	26.7 (1)
**2009**	516	265	129 (1.5)	3 (0.3)	1.7 (0.2)	818	25.9 (0.9)
**2010**	772	315	131 (1.8)	3.1 (0.4)	1.9 (0.1)	873	25.9 (0.6)
**2011**	633	346	130 (1.7)	2.3 (0.3)	1.8 (0.1)	994	26.3 (1.1)
**Total**	2823	951	-	-	1.8	4169	-
**Mean (SE)**	565 (76)	260 (37)	133 (0.9)	2.6 (0.2)	1.7 (0.1)	834 (52)	26.2 (0.2)

Between 2007 and 2011, there were a total of 2823 whale shark encounters ($\bar{\text{x}}$ = 564.6 ± 76 SE encounters per year, range = 309–772) by the ecotourism industry (Table 1). These encounters were of 951 individual whale sharks, of which 64 (6.7%) were female, 332 (34.9%) were male, and 555 (58.4%) were of indeterminate gender. On average, 260 individuals ± 37 SE were observed per year (range = 134–346). Most individuals (561, or 59%) were encountered only once between 2007 and 2011. Of the remaining sharks, 273 individuals were encountered between two and five times (28.7%), 63 were encountered between six and 10 times (6.6%) and 54 were encountered more than 10 times (5.7%). In total, 390 sharks were seen more than once (41%). The majority of sharks (748 or 78.6%) were encountered in only one of the five years of the study, with 110 (11.6%) encountered in two years, 57 (6%) in three years, 21 (2.2%) in four years, and 15 (1.6%) across the entire study period.

Each season typically began in late March and ended in the middle of July. Sharks were first encountered, on average, on the 132^nd^ (± 1 SE day) calendar day (the 13^th^ of May) each year (range = 67–248). The encounter period for the aggregation each year (the number of days between the first shark encounter and last each year) was relatively variable ($\bar{\text{x}}$ = 141.2 ± 14.5 days, range = 101–181) as was the mean within-season days between encounters was 2.6 ± 0.2 SE (range = 2.1–3.1) (Table 1).

Sea surface temperature (SST) averaged 26.2 ± 0.2°C across the study (Table 1) and did not vary significantly among years (ANOVA, p = 0.98, df = 4).

### Encounter trends for the Ningaloo aggregation

Re-encounters of whale sharks did not appear to be a function of either earlier exposure to tourists or the number of years a shark was encountered. For consecutive year pairs, on average, the total encounters per whale shark in year one were higher for whale sharks re-encountered in year two compared to those only encountered in year one, in terms of both overall ($\bar{\text{x}}$ = 3 encounters per year ± 0.5, vs. $\bar{\text{x}}$ = 2 encounters per year ± 0.3 respectively) and in each pair of years (2008–2009 $\bar{\text{x}}$ = 4.4 ± 0.5, vs. $\bar{\text{x}}$ = 1.8 ± 0.1; 2009–2010 $\bar{\text{x}}$ = 2.9 ± 0.3, vs. $\bar{\text{x}}$ = 1.5 ± 0.1; 2010–2011 $\bar{\text{x}}$ = 3.7 ± 0.4, vs. $\bar{\text{x}}$ = 1.9 ± 0.1). Welch’s t-tests indicated that the mean total encounters in year one for re-encountered individuals was significantly higher than for sharks that were not re-encountered in consecutive year pairs (2008–2009 p = 7.1e^-6^; 2009–2010 p = 7.7e^-6^; 2010–2011 p = 0.0002), with the exception of 2007–2008 when they did not differ (p = 0.11).

Mean encounters per year, per whale shark were positively correlated with the number of years the sharks were encountered(r^2^ = 0.92, p = 0.009; Fig 1A).

### Day of first encounter, days between encounters and residency time

**Fig 2:**
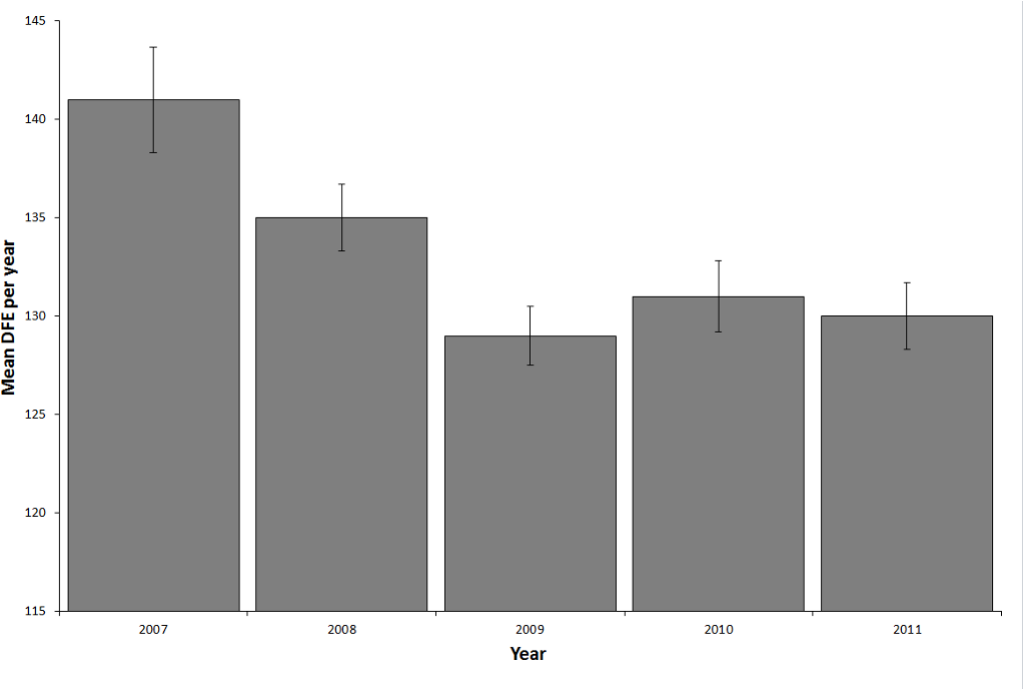
Variation in DFE (day of first encounter) per whale shark per year by year with standard errors calculated from individual whale sharks.

Mean day of first encounter in 2007 differed significantly from all other years (ANOVA, p = 0.0003, df = 4; Tukey’s HSD p<0.004, Fig 2). Mean DFE per whale shark was significantly (r^2^ = 0.91, p = 0.012) and negatively correlated to the number of years a shark was encountered (Fig 1B).

Mean days between encounters per whale shark did not differ among years (ANOVA, p = 0.18, df = 4), but was significantly and negatively correlated with the number of years a shark was re-encountered (p = 6.5e^-26^; Fig 1C). Furthermore, mean residency time was negatively correlated with the number of years a shark was encountered (p = 0.01, Fig 1D). For consecutive year pairs, on average, residency time in year two was higher for sharks encountered in year one compared to those only encountered in year two (2007–2008 $\bar{\text{x}}$ = 24.7 ± 3.7, vs. 2008 $\bar{\text{x}}$ = 17.2 ± 1.2; 2008–2009 $\bar{\text{x}}$ = 21.9 ± 3.1, vs. 2009 $\bar{\text{x}}$ = 7.3 ± 1; 2009–2010 $\bar{\text{x}}$ = 24.5 ± 3.5, vs. 2010 $\bar{\text{x}}$ = 10.2 ± 1.3; 2010–2011 $\bar{\text{x}}$ = 20 ± 3.4, vs. 2011 $\bar{\text{x}}$ = 5.3 ± 1). Welch’s t tests indicated that the mean residency time in year two was significantly higher for sharks encountered in year one in comparison to those only encountered in year two pairs (2007–2008 p = 4.7e-5; 2008–2009 p = 2.07e-5; 2009–2010 p = 0.0002, 2010–2011 p = 7.7e-5).

### Trends in encounters per boat trip

**Fig 3:**
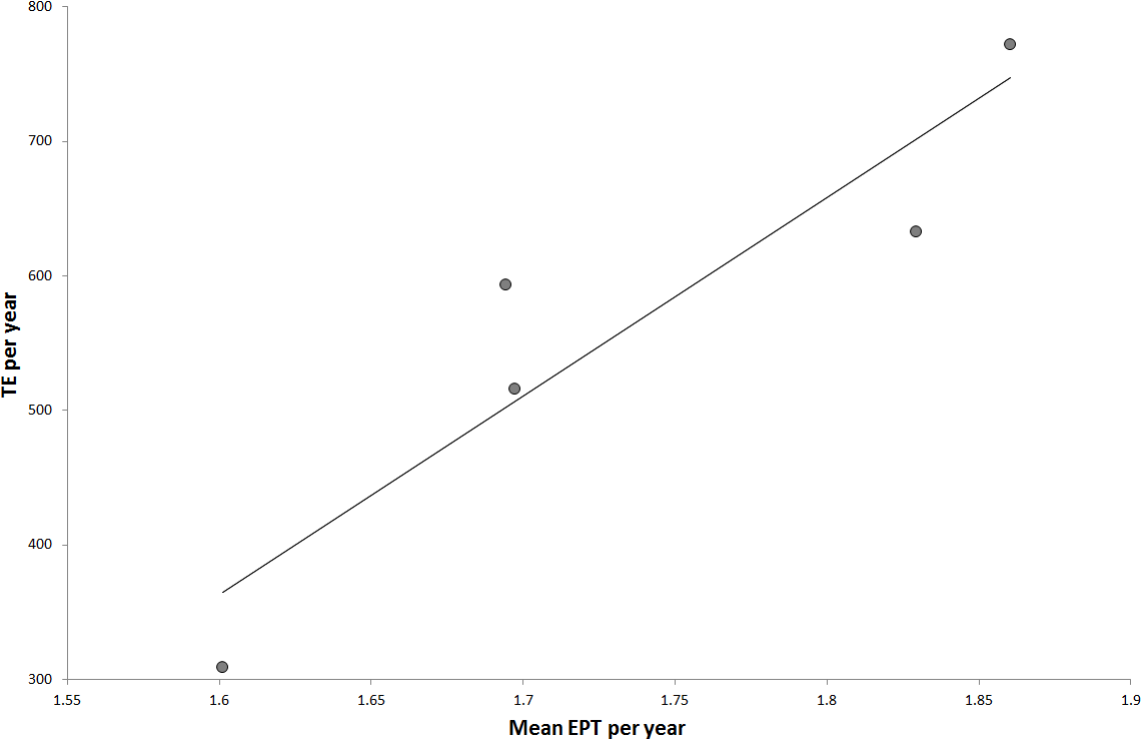
Relationships between TE (total encounters) per year and mean EPT (encounters per trip). TE year^-1^ = 1476EPT year^-1^–1999, R^2^ = 0.86, p = 0.046.

Encounters per trip were similar in all years (ANOVA, p = 0.26, df = 4) ranging between 1.9 (± 0.2 SE) in 2010 and 1.6 (± 0.2 SE) in 2007 (Table 1). Mean encounters per trip was positively correlated (r^2^ = 0.86, p = 0.045) with total encounters per year (Fig 3), but was not correlated with mean day of first encounter or days between encounters per year. Spotter plane hours varied little between years ($\bar{\text{x}}$ = 1263 ± 28 hours) and encounters per trip per year was not correlated with whale sharks encountered per spotter plane hour per year (regression analysis, p = 0.1).

**Fig 4:**
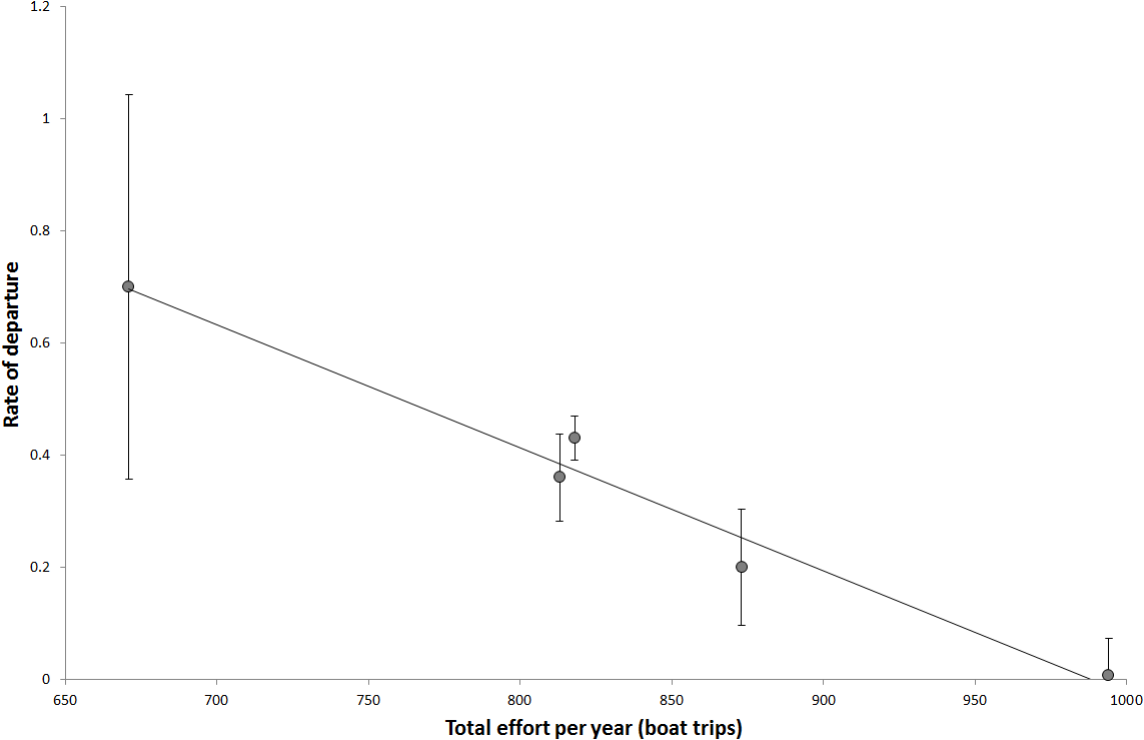
The rate of departure as a function of total effort ± standard errors indicating that the rate of departure from the aggregation slows with increasing numbers of trips. Rate of departure = -0.0022effort + 2.17, R^2^ = 0.97, p = 0.002.

Encounters per trip per year remained stable irrespective of variation in total effort (boat trips) per year (regression analysis, p = 0.4). The rate of departure from the aggregation was negatively correlated with total effort from 2007 to 2011 (r^2^ = 0.97, p = 0.002) (Fig 4).

### Effect of environmental variables

There were four and three correlated pairs of environmental variables for the yearly and monthly analyses respectively: sea surface temperature and sea level (0.86), SST and wind shear (-0.87), wind shear and sea level (-0.78), and SOI and chlorophyll *a* concentration (0.98) for the yearly analysis (Table 2), and SST and SL (0.81), SST and chlorophyll *a* concentration (-0.67), and chlorophyll *a* concentration and wind shear (0.63) for the monthly analysis (Table 2).

**Table 2:** Correlation matrix between the environmental variables of Southern Oscillation Index (SOI), sea surface temperature (SST), sea level (SL), wind shear, and chlorophyll *a* concentration from 2007 to 2011 both monthly and annually.

	SOI_mth_	SOI_yr_	SST_mth_	SST_yr_	SL_mth_	SL_yr_	Wind shear_mth_	Wind shear_yr_
**SST**	-0.05	0.50	-	-	-	-	-	-
**SL**	0.18	0.03	0.81	0.86	-	-	-	-
**Wind shear**	0.20	0.36	0.36	-0.87	-0.06	0.78	-	-
**Chlorophyll *a***	0.10	0.98	-0.67	-0.24	-0.25	0.22	-0.63	-0.43

**Fig 5:**
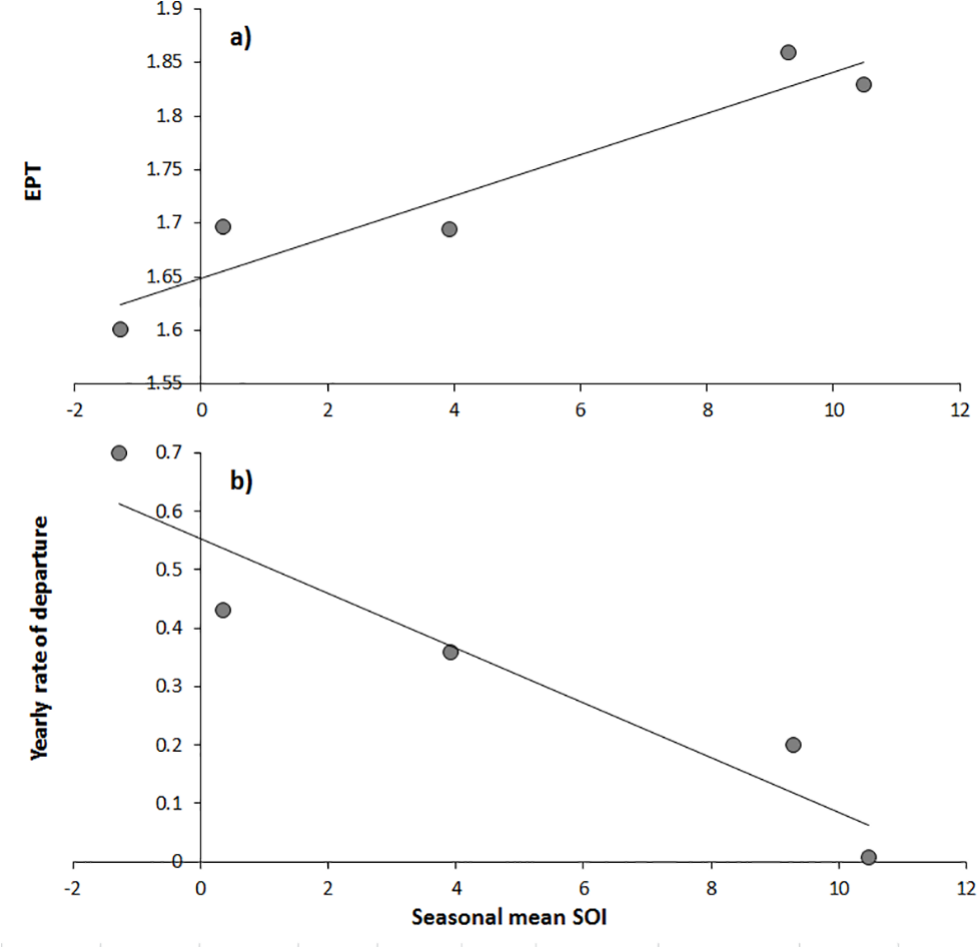
Relationships between yearly rate of departure from the aggregation and encounters per trip (EPT), and the seasonal mean Southern Oscillation Index (days between encounters). Slope = 0.05SST + 0.55, R^2^ = 0.89, p = 0.015 and EPT = -0.02SOI +1.65, R^2^ = 0.90, p = 0.014.

**Fig 6:**
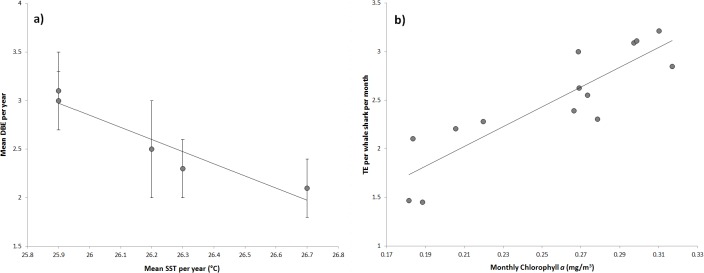
Relationships between a) mean DBE (days between encounters) and the mean SST (sea surface temperature) per year ± standard errors and b) total encounters (TE) per whale shark per month and the monthly concentration of chlorophyll *a*. 6a) Mean DBS = -1.3SST + 35.4, R^2^ = 0.90, p = 0.018; 6b) TE = 10.20CHL*a* – 0.12, R^2^ = 0.77, p = 3.34e^-5^.

Encounters per trip were highly correlated to mean seasonal SOI on a yearly basis (r^2^ = 0.90, p = 0.01, Fig 5A) as was the yearly rate of departure from the aggregation and mean seasonal SOI (r^2^ = 0.89, p = 0.01, Fig 5B). Additionally, mean days between encounters was positively correlated with mean SST per year (r² = 0.90, p = 0.01, Fig 6A).

**Fig 7:**
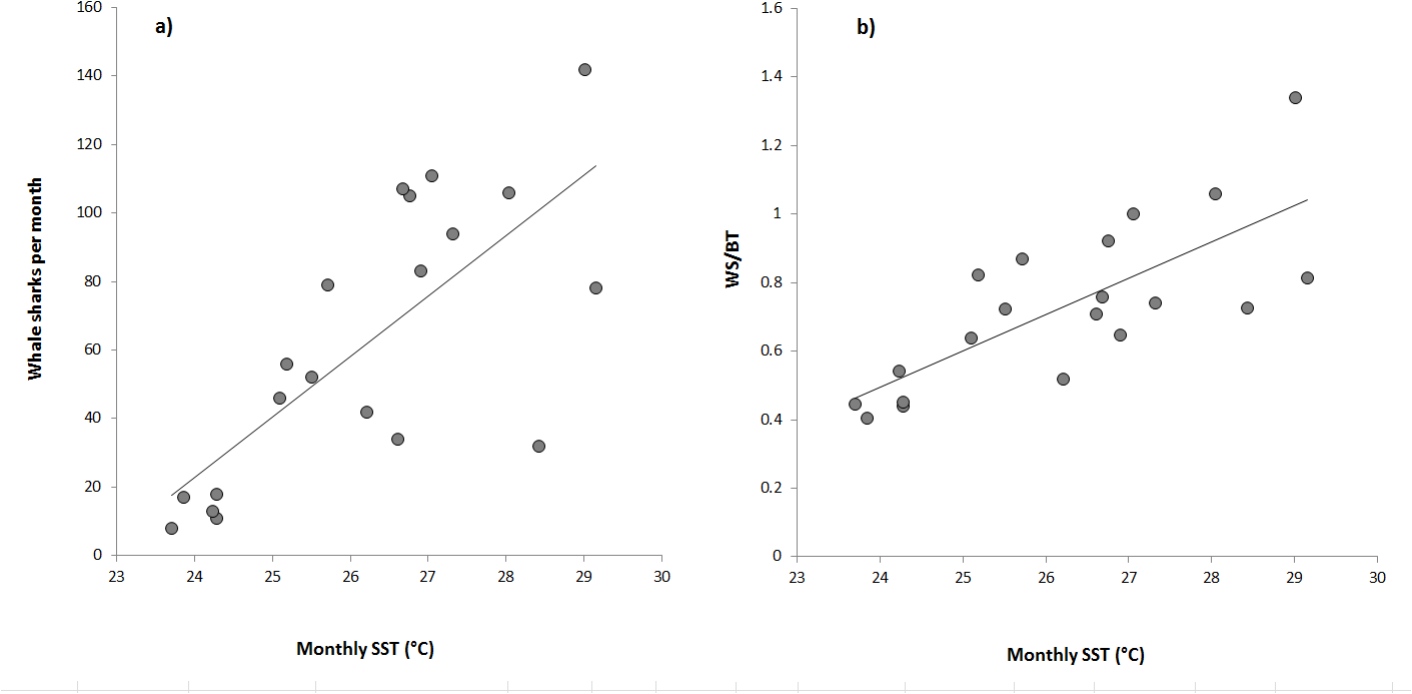
Relationships between whale sharks per month (WS), whale sharks per boat trip per month (WS/BT) and the monthly SST (°C). WS = 17.65SST – 400.69, R^2^ = 0.54, p = 0.0002 and WS/BT = 0.11SST – 2.05, R^2^ = 0.57, p = 0.0001.

For monthly data sets, total encounters per whale shark per month was positively correlated to chlorophyll *a* concentration (r² = 0.77, p = 3.34e^-05^, Fig 6B), the number of whale sharks encountered per month was positively correlated to SST (r² = 0.54, p = 0.0002, Fig 7), and whale sharks per boat trip per month was positively correlated to SST (r² = 0.57, p = 0.0001, Fig 7).

## Discussion

### Do ecotourism interactions impact the Ningaloo aggregation?

We found no evidence for long-term (multi-year) disruption of visitation patterns of whale sharks from ecotourism encounters. There were no negative trends through time in total encounters, day of first encounter, encounters per trip, residency time or the rate of departure (slope) with respect to the number of tourist encounters that animals were subjected to. Indeed, in years where the number of boat trips was high, whale sharks appeared to leave the aggregation at a slower rate. While this could simply reflect an increased number of boat trips leading to higher encounter rates, this is unlikely given that encounters per trip were constant across increasing numbers of trips and there was no evidence of technological creep. Finally, there was no evidence that high exposure to ecotourism interactions led to inter-annual decreases in total encounters per year, an increase in days between encounters, or a later day of first encounter.

Rather than a decline in encounter and visitation patterns, we found that in consecutive year pairs of sightings, returning whale sharks had higher mean total encounters than non-returning whale sharks in the previous year. Furthermore, returning sharks had a longer residency time in the year two than sharks that were only encountered in year two and mean days between encounters were negatively correlated to the number of years a shark was encountered. These results might indicate that individuals returning to Ningaloo Reef become accustomed to encounters with tourists. While there is evidence of sharks becoming habituated in this manner, this generally occurs with predatory sharks in situations where food is provisioned in order to aggregate them for viewing by tourists. This may have negative impacts, including increasing energy costs for the animals [43–44]. However, provisioning of whale sharks does not occur at Ningaloo, although this is now happening at localities in Indonesia and the Philippines [15]. Whatever the underlying reason for higher encounters of returning sharks compared to non-returning individuals, our results suggest there is little difference in visitation rates for newly encountered sharks and re-encountered individuals and that in all likelihood, prior encounters are having little effect on future visitation rates. These results are supported by mark-recapture analyses of the Ningaloo whale shark aggregation that have shown that there is no change in capture efficiency of whale sharks across years and that the probability of capture of unmarked or new whale sharks is the same as the probability of capture for marked individuals-[45–46].

Our analysis suggested that variability in oceanographic and atmospheric variables had a far greater effect on whale shark visitation at Ningaloo than did tourism interactions. The SOI had an important influence on the annual whale shark aggregation with sharks leaving Ningaloo more slowly and being encountered more often per boat trip in years when the index was high (>4). Additionally, increasing mean sea surface temperature (SST) led to a reduction in the mean number of days between encounters per whale shark per year. Within seasons, our monthly analysis suggested that more whale sharks were encountered by the tourist industry, per month and per boat trip per month, when SST was high. Furthermore, increases in total encounters per whale shark per month were associated with higher levels of chlorophyll *a* in the water.

Our findings support earlier studies that have demonstrated the whale shark aggregation at Ningaloo is influenced by oceanographic and atmospheric processes and biophysical variables [32,41]. For example, Wilson et al. [41] found that abundance of whale sharks at Ningaloo was higher in La Niña than El Niño years and modelling by Sleeman et al. [32] indicated that the SOI explained the most deviance in whale shark abundance on a weekly basis during the peak of the season. SOI is a measure of the El Niño-Southern Oscillation (ENSO), which influences the strength of the Leeuwin Current and sea level along the Western Australia coastline [47]. During a La Niña year, the strength of the Leeuwin Current increases, driving patterns of higher productivity along the central WA coast. Conversely, current strength and productivity decline in El Niño years. Sleeman et al. [32] suggested that these changes in productivity associated with the ENSO phenomenon could drive changes in the abundance and residency patterns of sharks along the WA coast and at Ningaloo. Indeed, there is evidence that SOI drives changes in abundance in other shark species with Towner et al [48] finding the abundance of white sharks in Gansbaai, South Africa was strongly linked to inter-annual changes in this metric.

Chlorophyll *a* and SST were important influences on trends of within-season encounters at Ningaloo. This result is not surprising given the well-established link between chlorophyll *a* and zooplankton biomass in marine ecosystems [49–50] and the evidence that whale sharks aggregate at Ningaloo to feed on zooplankton [32,51–52]. Both chlorophyll *a* and SST are thought to influence the distribution of planktivores and secondary carnivores that feed on species at lower trophic-levels such as zooplankton, krill and small bait fishes [53–54], although the relationship between these variables may be complex. We found that chlorophyll *a* and SST were negatively correlated (0.67; Table 2), so that the number of whale sharks encountered per month increased with increasing SST, while the TE per whale shark per month declined as chlorophyll *a* concentration declined. Assuming that chlorophyll *a* concentration is a reasonable proxy for zooplankton abundance (at least at this spatial and temporal scale), these patterns could be explained by changes in the feeding modes of whale sharks at different concentrations of zooplankton. At high densities, whale sharks will restrict their movements and focus feeding on zooplankton prey patches, similar to other filter-feeding sharks such as the basking shark [55–56] and other marine megafauna such as seals [57] and whales [58]. In contrast, at low zooplankton densities whale sharks may spend more of their time ram filter-feeding and searching for prey, so that they cover a larger area. Our ongoing analyses of tracking data suggest that such patterns of restricted area search (i.e. the concentration of foraging effort in areas with high prey encounter rates) [59] are typical of whale shark behaviour at Ningaloo Reef (M. Meekan unpublished data). These contrasting movement patterns probably have consequences for the tourism industry since sharks remaining in one area would be far easier to locate repeatedly than those traveling in a search mode. Behaviours such as restricted area search thus provide a plausible explanation as to why total encounters per whale shark per month should decline as chlorophyll *a* concentration decreases.

We cannot exclude the possibility that those sharks that were only encountered once during the study were not re-encountered in following years because they avoided any further interactions with tourism vessels after the initial encounter. However, this seems unlikely given that we saw little evidence of changes in terms of total encounters, day of first encounter, and days between encounters over the course of the study, and SST and SOI appeared to influence visitation by sharks far more than any of our disturbance metrics. Furthermore, such a relationship would also require the unlikely possibility that individual sharks at Ningaloo display one of two distinct behavioural modes: either apparent unconcern towards interactions or complete avoidance after an initial encounter. A more parsimonious explanation is that sharks in this population vary in their degree of transience and site fidelity, which is a common phenomenon both in other sharks [60] and species such as whales [61] and dolphins [62]. Alternatively, or in addition, in any given year the aggregation at Ningaloo may represent only a portion of a regional super-population containing sharks that visit the reef at some point during their lives [26]. Finally, the sharks encountered by ecotourism operators may only represent a small fraction of the entire aggregation that can be accessed by operators at any one time given the large spatial scale of the aggregation site (approximately 250 km of coastline).

It is unlikely that ecotourism operations are affecting breeding or foraging behaviours. Previous reports show that the aggregation at Ningaloo is mostly made up of juvenile males between three to eight metres in total length [26,36] and our results were consistent with this finding, with 84% of the identified individuals encountered in this study found to be males. In such an aggregation, it is unlikely that ecotourism interactions could directly affect reproduction. Although it does occur, feeding behaviour is rarely witnessed at the surface during daytime and is most common at dusk [51,63–65]. Given that most tourist operations cease in the late afternoon (usually not later than 4.00pm), this reduces the likelihood that ecotourism interactions during the daytime at Ningaloo Reef disrupt localised foraging behaviour.

There is evidence to suggest that short-term (minutes to hours) behaviours including rolling, banking and diving by whale sharks in response to approaches by divers and boats are negative reactions to the presence of humans [15]. Indeed, a study of whale sharks at Donsol in the Philippines demonstrated that sharks change direction when confronted by boats and swimmers, dive when disturbed during feeding, and shudder when touched by snorkelers [15]. However, observations from Ningaloo indicate that whale sharks do exhibit these behaviours naturally. Gunn et al. [66] and Colman [10] argued that until the natural behaviour of whale sharks is better understood, the extent to which tourism drives such short-term behaviours cannot be conclusively determined. Our analysis did not seek to determine the impacts of encounters on the immediate behaviour of whale sharks, but rather how encounters impacted annual visitation to Ningaloo Reef. To this end, we cannot discount the possibility that these encounters have a detrimental impact on immediate behaviour of whale sharks at Ningaloo. Approaches such as those employed in behavioural studies of cetaceans [67–68] would be appropriate for examining this possibility at Ningaloo Reef. However, our study suggests that whatever the short-term reaction of sharks to tourism encounters, there is little evidence for these having any adverse long-term effect on the residency or return of sharks to Ningaloo.

Given that the total encounters of whale sharks were expected to increase along with numbers of boat trips, we also analysed total encounters per trip as a function of the number of trips to account for sampling effort. Total encounters per trip were stable with respect to total effort (boat trips) and an increase in the number of whale sharks per year, suggested that increasing the number of boat trips did not appear to reduce encounters or whale shark numbers. We also considered the possibility of technological creep masking potential declines in whale shark numbers, however, our analysis of spotter plane hours showed no significant increase in the efficiency of the industry across the study period despite an increase in numbers of whale sharks across years.

### Implications for the assessment of inter-annual disturbance and ecotourism management

Our approach is broadly applicable to the study of the effects of ecotourism encounters and environmental parameters for any species where multi-year databases that individually identify animals are available for analysis. While other studies have utilised encounter records for mark-recapture analysis of population numbers and survival rates [26,36], analysis of species habitat and distribution [69], and studies of direct behavioural responses to interactions [67], our study provides a template for using multi-year encounter records to analyse visitation in response to ecotourism interactions and environmental factors in the absence of control sites and behavioural data. Potential species for such an analysis could include grey nurse sharks (*Carcharias taurus*) [70], manta rays (*Manta alfredi*) [71] and cetaceans [67]. For these animals, large photo-identification data sets have also been compiled over multiple years, although the factors likely to influence or confound these data bases will differ from those in our study.

Our results suggest that environmental factors at Ningaloo Reef have a greater influence on whale shark visitation than exposure to tourism. We found no evidence of negative impacts of tourism on whale shark visitation on a long-term basis. Our analysis was based on a large aggregation area governed by a management regime that limits the number of operator licenses at Ningaloo and monitors operator compliance to a code of conduct. Our results may not be directly applicable to other aggregation sites where licenses are not controlled and a code of conduct is not monitored for compliance. Given that the popularity of shark diving is increasing rapidly and that provisioning in order to promote aggregations at predictable times and places is becoming a feature of many of these operations (now even in the case of whale sharks), studies of a similar temporal scope and under similar controls at other locations are essential in order to ensure that the desire of the public to view these animals in their natural habitat does not compromise their well-being.
